# Clinical Outcomes from Dose-Reduced Radiotherapy to the Prostate in Elderly Patients with Localized Prostate Cancer

**DOI:** 10.3390/curroncol28050318

**Published:** 2021-09-26

**Authors:** Nina Samson, Rutvij A. Khanolkar, Sarah Quirk, Harvey Quon, Michael Roumeliotis, Alex Balogh, Michael Sia, Kundan Thind, Siraj Husain, Kevin Martell

**Affiliations:** 1 Department of Oncology, University of Calgary, Calgary, AB T2N 4N2, Canada; nina.samson@ahs.ca (N.S.); rutvij.khanolkar@ahs.ca (R.A.K.); harvey.quon@ahs.ca (H.Q.); alex.balogh@ahs.ca (A.B.); michael.sia@ahs.ca (M.S.); siraj.husain@ahs.ca (S.H.); 2 Cumming School of Medicine, University of Calgary, Calgary, AB T2N 4N1, Canada; sarah.quirk@ahs.ca (S.Q.); michael.roumeliotis@ahs.ca (M.R.); kundan.thind@ahs.ca (K.T.); 3 Department of Physics and Astronomy, University of Calgary, Calgary, AB T2N 1N4, Canada

**Keywords:** prostate cancer, elderly, treatment, palliation

## Abstract

Radical treatment of localized prostate cancer in elderly patients may lead to unacceptable treatment-associated toxicities that adversely impact quality of life without improving survival outcomes. This study reports on a cohort of 54 elderly (>70 years) patients that received 4000–5000 cGy of palliative external beam radiotherapy (EBRT) as an alternative to androgen deprivation therapy (ADT). The primary outcome of interest was the period of ADT-free survival, and secondary outcomes included overall survival (OS) and metastases-free survival (MFS). Kaplan–Meier regression was used to estimate survival outcomes. Thirty-six (67%) patients achieved a break in ADT post-radiotherapy, with a median time to ADT reinitiation of 20 months. Common Terminology Criteria for Adverse Events (CTCAE) were limited to low-grade gastrointestinal (GI) or genitourinary (GU) toxicities, with no skin toxicities observed. Grade 1 GI toxicity was observed in 9 (17%) patients, and grades 1 and 2 GU toxicities were observed in 13 (24%) and 3 (6%) patients, respectively, with no higher-grade toxicities reported. Five-year MFS and OS were 56% and 78%, respectively. In summary, the treatment regimen was well-tolerated and achieved durable ADT-free survival in most patients. Dose-reduced EBRT appears to be a viable alternative to ADT in elderly patients with localized prostate cancer.

## 1. Introduction

Prostate cancer is among the most common malignant neoplasms and causes of cancer death in North America [1,2,3]. Prostate cancer is often first identified on screening due to widespread implementation of prostate-specific antigen (PSA)-based screening, and trans-rectal ultrasound (TRUS) guided tissue-biopsies are used to confirm diagnoses [4].

A common clinical scenario encountered by oncologists is a new diagnosis of prostate cancer within an elderly patient (>70 years old). These patients often present with locally advanced disease which leads to increased risk of adverse outcomes including urinary incontinence, renal failure or obstructive symptoms [5,6,7,8]. Otherwise, in these patients, the impact of radical therapy on long-term survival is likely limited [9,10]. Radical external beam radiotherapy (EBRT) is associated with adverse short-term side effects including desquamation, urinary obstruction, incontinence, and diarrhea, as well as adverse long-term side effects such as bowel and bladder dysregulation, urethral stricture formation, and radiation cystitis/proctitis in severe cases [11]. Due to this, elderly patients are often managed with androgen deprivation therapy (ADT), which aims to shrink or reduce tumor growth by depriving prostate cancer of androgens such as testosterone and dihydrotestosterone that normally drive cellular survival and proliferation [12]. Despite its efficacy, ADT is also associated with toxicities such as sexual or urinary dysfunction, decreased mineral bone density, hot flashes, and potentially, cognitive or cardiac decline [13,14]. Therefore, elderly patients with prostate cancer may stand to substantially benefit from the implementation of therapeutic options that improve survival while still preserving quality of life.

In contrast to dose-escalated curative-intent therapy (e.g., EBRT doses of 7800 cGy in 39 fractions or 6000 cGy in 20 fractions), radiotherapy with doses of up to 5000 cGy to the pelvis is generally well-tolerated, making it a feasible alternative in elderly patients with prostate cancer [15]. At the current study’s institution, it has been common practice to offer elderly patients high-dose palliative radiotherapy consisting of up to 5000 cGy delivered across 20 fractions with the aim of controlling local disease, delaying progression, and achieving a reduction or pause in ADT. The present study’s objective was to report on toxicities associated with this treatment strategy, the long-term outcomes, and its efficacy at achieving a pause in ADT.

## 2. Materials and Methods

### 2.1. Study Design and Patient Selection

**Figure 1:**
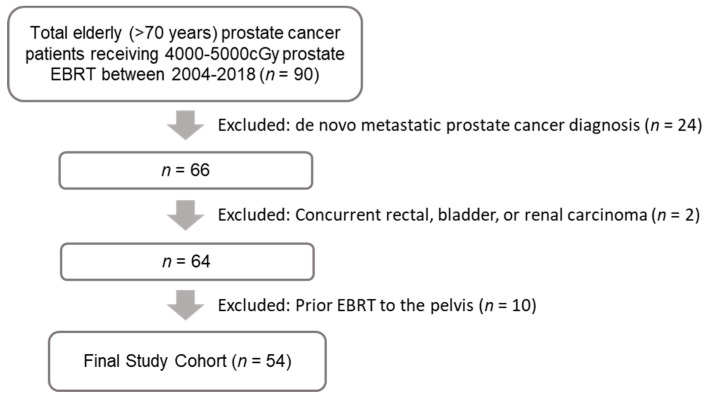
Flow diagram with number of patients excluded from analysis and reasons for their exclusion.

After institutional ethics approval, the AriaRO Unified Reporting Application interface (Varian, Palo Alto, CA, USA) was used to identify 90 patients with birthdate(s) on or before 1 September 1948 who received radiotherapy treatment(s) with doses between 4000 and 5000 cGy to the prostate between 1 January 2004 and 31 December 2018. Exclusion criteria included patients who had prior radical therapy for prostate cancer, concurrent diagnoses of rectal, bladder, and/or renal cell carcinoma, de novo metastatic prostate cancer diagnosis, prior radiotherapy to the pelvis for any reason or having received EBRT to both the prostate and pelvic lymphatics (Figure 1). A retrospective chart review of both the radiotherapy treatment planning system and the institutional electronic medical record which contains all staging and follow-up information as well as any visitations to external hospitals or bloodwork/imaging conducted within the healthcare jurisdiction was conducted. Fifty-four patients meeting the inclusion criteria for the study were identified.

### 2.2. Treatment Regimen(s) and Rationale(s)

With regard to practices surrounding management recommendations, the institutional practice over the study period was to recommend ADT for elderly patients with new diagnoses of prostate cancer. However, at the radiation oncologist’s discretion, a variation in practice to offer dose-reduced localized EBRT to the prostate was accepted as an option. Neither brachytherapy nor radical prostatectomy was routinely considered a recommended management option for these patients over the study period. When dose-reduced radiotherapy was offered, treatments were typically inverse planned using static field sliding window intensity modulated radiotherapy (IMRT) or volumetric modulated radiotherapy (VMAT). Organ at risk dose constraints were not standardized institutionally for these dose-reduced treatments and were provided by the individual radiation oncologist on a patient-specific basis. In general, constraints achieved were more conservative than those of published hypofractionation trials [16,17]. At treatment delivery, routine practice until 2013 was to match boney anatomy using orthogonal kV films. After 2013, matches were performed based on assessment of the prostate–rectal interface as seen on cone beam computer tomography (CT) imaging. 

In cases where dose-reduced radiotherapy was offered, the treatment intent was to either avoid initiating, discontinue, or provide a break in ADT treatment. The rationale for this was typically to avoid or provide respite in side effects. In such cases, follow-up was routinely lifelong in 3–6-month intervals with the primary radiation oncologist and/or medical oncologist treating the patient. At each follow-up visit, PSA, American urological association (AUA) symptom assessments and toxicity were evaluated.

### 2.3. Data Collection

The radiotherapy data for all patients included in this study was extracted directly using the AriaRO Unified reporting application interface. Specifically, dose-volume-histogram values for the body, rectum, bladder and planning-target-volume (PTV) were exported and amalgamated into a single database. Treatment start and end dates, doses and fractionations were all available in this record. For retrospective chart reviews, the patient’s electronic medical record and the jurisdictional electronic health record were reviewed for all patients included in the study. Systematically structured pathology reports, as dictated by dedicated genitourinary pathologists were used to extract all pathological information. All CT reports, bone scan reports, and consultation notes were reviewed for clinical staging information. PSA values were reviewed to determine the pre-treatment PSA, the PSA nadir, the first post-RT PSA >0.5 ng/mL, the first post-RT PSA >10.0 ng/mL, and the corresponding measurement dates. In addition, the start and end dates of all hormone administration(s) were collected (these were available in the jurisdictional electronic health record). Finally, for survival and toxicity outcomes, the electronic medical and electronic health records were reviewed. Charts were reviewed for radiographic confirmation of systematic spread of disease, follow-up notes (in intervals as above), AUA symptom assessments, death, or any presentation to any acute care facility (researchers focused on presentation for possible radiotherapy toxicity). All potential toxicities were reviewed at the time of follow-up by the attending radiation oncologist to determine if it was related to radiotherapy. If a comment on whether the toxicity was attributable to radiotherapy was not found in the follow-up note, an independent radiation oncologist reviewed the event and determined causation. If causation could not be determined confidently, it was assumed as due to radiotherapy.

### 2.4. Study Endpoints

The primary study outcome was the rate and duration of ADT free period following radiotherapy. Secondary outcomes included the rates of common terminology criteria for adverse events reporting (CTCAE) version 5.0 grade 3 genitourinary (GU) and gastrointestinal (GI) toxicity, PSA response to treatment, and metastasis-free and overall survival [18]. 

### 2.5. Statistical Considerations

Descriptive statistics were used to characterize the cohort. For continuous variables median (interquartile range) and for categorical variables numbers (%) were used for description purposes. PSA nadirs were calculated as the lowest PSA taken at least 6 months post-radiotherapy in patients on continuous ADT and at least 6 months after completion of ADT (to allow for testosterone recover) in patients who did not receive continuous androgen blockade. For estimations of survival outcomes, the Kaplan–Meier method was used. Metastasis-free-survival was calculated as from the time of diagnosis to the time to radiographic evidence of metastatic disease or death and censured to last follow-up. Overall survival was calculated as time to death and censured to last follow-up. All analyses were performed using the R programming language version 4.3.1 (www.r-project.org).

## 3. Results

### 3.1. Baseline Patient and Tumor Characteristics

Fifty-four patients meeting the inclusion criteria for the study were identified. No patients were lost to follow-up. Baseline patient demographics are summarized in Table 1. In total, 53 (98%) patients had pathologic confirmation of prostate cancer with TRUS-guided biopsy. One (2%) patient refused the biopsy and was treated based on elevated PSA and abnormal digital rectal examination (DRE), with absence of metastatic disease on work-up (negative staging CT and bone scan). Median time from pathologic diagnosis of prostate cancer to administration of radiotherapy was 13 (13–31) months.

### 3.2. Radiotherapy Treatment Characteristics

Radiotherapy treatment parameters are reported in Table 2. Two (4%) patients with unilateral hip replacements were treated with 3-field 3D conformal radiotherapy (3D-CRT), 9 (17%) patients were treated using 4-field box plans. One (2%), 1 (2%), and 41 (76%) patient(s) were treated using 5-field IMRT, 7-field IMRT and VMAT plans, respectively. Within the cohort, the minimum PTV coverage was 99.5% of the volume receiving 95% of the prescribed dose. The three patients with the greatest percentage of bladder tissue receiving 50 Gy (equivalent dose in 2 Gy fractions with an α/β of 2-EQD2Gy2) had 40%, 27% and 22% of the bladder receiving 50 Gy (EQD2Gy3). 

### 3.3. Androgen Deprivation Therapy

No (0%) patient received systemic therapy other than leuprolide and/or bicalutamide prior to radiotherapy. Fifty-two (96%) patients had ADT for at least one time point before, during or immediately following their radiotherapy with a median duration of ADT of 24 (12–34) months. Fifty patients (93%) received ADT prior to radiotherapy for a median of 8 (5–17) months. Of these, three patients were on a break from ADT at the time of RT. Times from the end of their last ADT to radiotherapy were 10, 15, and 36 months. The patient receiving a 36-month break received 1 year of ADT adjuvantly with their radiotherapy. The patient receiving a 10-month break required no further ADT.

Sixteen (30%) patients received continuous ADT surrounding their radiotherapy. In the 36 (67%; two patients did not receive any ADT) patients who did not receive continuous ADT, median duration of combined neoadjuvant and adjuvant ADT was 18 (10–25) months. Overall, 5 of 38 (13%) patients restarted or started ADT after having received radiotherapy. Median time to re-initiation of ADT was 20 (13–23) months. Ten (19%) patients received only neoadjuvant ADT. Of these 10, 3 (30%) patients restarted ADT within the study period. Time to restarting ADT was 13, 23, and 83 months from the end of adjuvant radiotherapy in these patients. One patient (2%) received 33 months of neoadjuvant/adjuvant ADT and then restarted ADT 20 months later. No other patient receiving neoadjuvant/adjuvant ADT re-initiated ADT.

### 3.4. Genitourinary and Gastrointestinal Toxicity

Prior to radiotherapy, median AUA symptom score across the patient cohort was 7 (3–16). Pre-radiotherapy trans-urethral resection of the prostate (TURP) was performed on 19 (35%) of patients. In total, 14 (26%) patients were on tamsulosin; three (6%) of which were also on dutasteride prior to radiotherapy.

**Figure 2:**
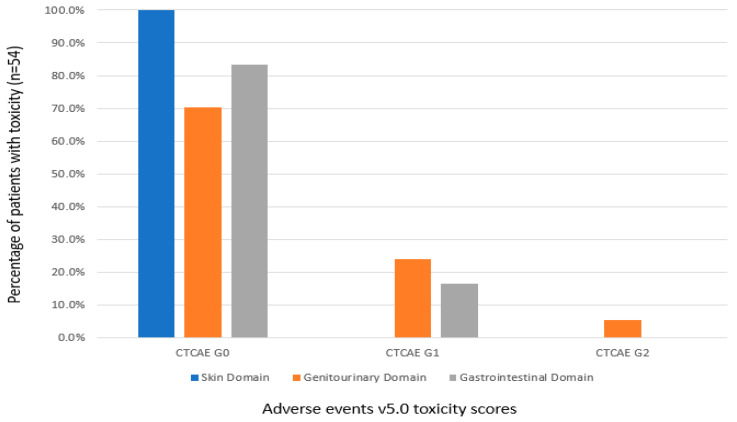
Maximum common terminology for adverse events v5.0 (CTCAE v5.0) toxicity scores at any time post-radiotherapy in the skin (blue), genitourinary (orange) and gastrointestinal (grey) domains.

After completion of radiotherapy, median AUA symptom score was 9 (4–15). Seventeen (32%) patients used tamsulosin post-radiotherapy. Post-radiotherapy CTCAE toxicity scores are depicted in Figure 2. No patient encountered CTCAE grade 3 or higher toxicity. Median number of post-radiotherapy presentations was 1 (0–2) in the cohort. Evaluation of all emergency department presentations revealed only 6 out of 89 (7%) total post-radiotherapy emergency room presentations were for genitourinary issues. Median number of presentations related to GI or GU issues was 0. Furthermore, no patient (0%) required post-radiotherapy TURP or urethral dilation. Five (9%) patients did undergo post-radiotherapy cystoscopy for mild hematuria.

### 3.5. PSA Response to Treatment

PSA response after radiotherapy is summarized in Table 3. Median PSA at 3 months post-radiotherapy was 0.2 (0.1–1.1) ng/mL. Overall, 50 (93%) patients had a PSA response to radiotherapy. Median PSA nadir post-treatment was 0.2 (0.1–1.2) ng/mL. Median time to PSA nadir was 5 (1–7) months. In the 38 (70%) patients who did not receive continuous ADT, 37 (97%) patients had a PSA response to radiotherapy, with median PSA nadir of 0.1 (0.1–0.4) ng/mL, and median time to PSA nadir was 5 (1–7) months.

Twenty-nine (54%) patients either had a post-radiotherapy PSA never decrease below or had subsequent post-radiotherapy PSAs rise to above 0.5 ng/mL. Within this group, median time to PSA > 0.5 ng/mL was 15 (2–24) months from completion of radiotherapy. Fourteen (26%) patients had a post-radiotherapy PSA rise above 10.0 ng/mL. Within this group, median time to PSA > 10 was 12 (1–17) months from completion of radiotherapy.

### 3.6. Metastasis-Free and Overall Survival

**Figure 3:**
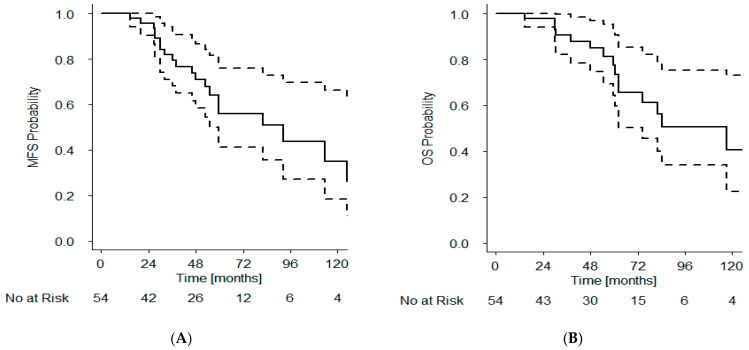
Kaplan–Meier estimate (solid line) and 95% confidence interval (broken lines) for MFS (**A**) and OS (**B**) for cohort of 54 patients receiving high-dose palliative radiotherapy to prostate.

A total of 13 (24%) patients developed metastatic disease. Kaplan–Meier estimated 5-year metastasis-free survival (MFS) was 56% (41–76) as shown in Figure 3A. Eighteen (33%) patients died after radiotherapy. Kaplan–Meier estimated 5-year overall survival was 78% (64–93) (Figure 3B).

## 4. Discussion

This study is the first report on the use of dose-reduced radiotherapy in elderly patients with prostate cancer. Outcomes were well annotated and without loss to follow-up. Overall, dose-reduced radiotherapy of 4000–5000 cGy to the prostate was well-tolerated. In total, 67% of patients who were on ADT had a treatment break after radiotherapy with a typical duration of 1.5 years.

The treatment of elderly patients with prostate cancer poses unique clinical challenges. Within this demographic, patients are more likely to have co-morbidities that decrease anticipated survival. For low-risk disease, the most common management strategy is watchful waiting [19,20,21]. This is well corroborated in a study by Bill-Axelson et al. where benefits of local therapy (prostatectomy) as compared to watchful waiting (with 67% of which eventually having ADT) had no statistically significant impact on overall survival, death from prostate cancer, or metastasis-free survival in patients over 65 years old [19,22]. However, while prostatectomy led to a significant reduction in ADT use, it also led to an increase in incontinence and impaired erectile function. Although there is no comparator arm in the present study, the reduction in ADT use experienced by patients initially on ADT supports this finding. 

This finding is important as androgen deprivation therapies have been associated with cardiac mortality [23,24,25]. In particular, in a study by D’Amico et al., the addition of just 6 months of ADT to external beam radiotherapy resulted in significantly increased mortality (hazard ratio 0.36 favoring avoidance of androgen deprivation) [14]. Although newer agents carry less cardiac risk, they are relatively expensive and often unapproved in patients without pre-existing cardiac morbidity [26,27]. Beyond this, patients on these treatments still endure other significant toxicities, with cognitive dysfunction, vasomotor symptoms, sarcopenia and decreased bone mineral density often being the most bothersome. With regard to the latter, sarcopenia has been associated with decreased quality of life and poorer survival [28,29].

Within this study the use of radiotherapy resulted in no grade 3 or higher CTCAE toxicity in skin, GI, or GU domains. This result is corroborated by emergency room presentations related to GI or GU concerns, with no further need for surgical intervention (e.g., TURP) and limited need for procedural intervention (i.e., cystoscopy). There was a slight increase in AUA symptom score reporting, as well as use of alpha-agonists post-radiotherapy. However, these differences are unlikely to be clinically significant. Furthermore, without radiotherapy, worsening urinary dysfunction and need for medical or procedural intervention is likely inevitable with local progression. 

The results of this study should be interpreted with caution as there are several limitations to it beyond its retrospective nature and the likelihood of inherent bias by design. The variability in tumor characteristics and PSA at the time of diagnosis meant there was variability in duration of ADT. Otherwise, this study is at high risk of selection bias for both the decision to initiate radiotherapy treatment and for the dose selected. Although the PSA kinetics post-radiotherapy suggest good efficacy of treatment, there was no firm criteria oncologists standardly followed for re-initiating ADT post-treatment or restaging patients. Given this, it is possible that the time to re-initiation of ADT or metastatic disease may be different than the experiences of other physicians should they adopt this protocol. Finally, this study only analyzed outcomes as related to external beam radiotherapy in elderly patients. Other treatment modalities with known low toxicity rates such as brachytherapy might be a preferred approach. This could be explored in future prospective studies.

Despite these limitations to the present study, the administration of dose-reduced local radiotherapy to the prostate may allow select elderly patients breaks in ADT use with delays to re-initiation or perhaps avoidance of ADT completely. It appears to be a safe, non-invasive option that can be considered by treating oncologists.

## Figures and Tables

**Table 1:** Baseline demographic and pathologic characteristics for 54 elderly patients with prostate cancer receiving dose-reduced, palliative external beam radiotherapy to the prostate.

	Median (IQR) or Number (%) *n* = 54
Age (years)	80 (76–83)
Pretreatment PSA (ng/mL)	21 (8–45)
Clinical stage	
T1b	4 (7%)
T1c	16 (30%)
T2a	7 (13%)
T2b	3 (6%)
T2c	5 (9%)
T3a	11 (20%)
T3b	1 (2%)
T4	7 (13%)
Method of pathologic diagnosis *	
TURP alone	13 (25%)
TRUS biopsy	40 (75%)
Grade group *	
1	3 (6%)
2	8 (15%)
3	10 (19%)
4	8 (15%)
5	24 (45%)
Cores sampled	12 (11–12)
Cores positive	7 (5–10)
% core tissue positive (%)	40 (17–54)
Pathologic peri-prostatic fat invasion	14 (30%)
NCCN risk grouping	
Favorable intermediate risk	2 (4%)
Unfavorable intermediate risk	6 (11%)
High risk	19 (35%)
Very high risk	27 (50%)

* One patient was treated on the basis of elevated PSA and abnormal DRE without pathological confirmation of disease.

**Table 2:** Radiotherapy treatment characteristics for 54 elderly patients with prostate cancer.

	Median (IQR) or Number (%) *n* = 54
Treatment technique	
3D-CRT	11 (20%)
IMRT	2 (4%)
VMAT	41 (76%)
Dose/Fractionation	
4000 cGy in 15 fractions	3 (6%)
5000 cGy in 20 fractions	51 (94%)
PTV dosimetry	
V95% [%]	100 (100–100)
D1cc [%]	105 (104–105)
D0.03cc [%]	106 (104–106)
Body D0.03cc [EQD2Gy3]	59 (58–60)
Bladder dosimetry	
V45Gy (EQD2Gy3) [%]	10 (7–15)
V50Gy (EQD2Gy3) [%]	8 (5–12)
V55Gy (EQD2Gy3) [%]	3 (2–6)
V60Gy (EQD2Gy3) [%]	0 (0–0)
Rectum dosimetry	
V45Gy (EQD2Gy3) [%]	18 (12–25)
V50Gy (EQD2Gy3) [%]	14 (9–19)
V55Gy (EQD2Gy3) [%]	0.9 (0.2–2.6)
V60Gy (EQD2Gy3) [%]	0 (0–0)

**Table 3:** Post-treatment prostate specific antigen.

	Median (IQR) or Number (%) *n* = 54
PSA value at 3 months (ng/mL)	0.2 (0.1–1.1)
Any PSA decline post-radiotherapy	50 (93%)
PSA nadir post-radiotherapy (ng/mL)	0.2 (0.1–1.2)
Time to PSA nadir (months)	5 (1–7)
Post-radiotherapy PSA >0.5	29 (54%)
Time to PSA >0.5 (months)	15 (2–24)
Post-radiotherapy PSA >10.0	13 (24%)
Time to PSA >10.0 (months)	12 (1–17)

## Data Availability

Data are only housed on institutional servers as per ethics policy.
